# Non-linear transformation of enzyme-linked immunosorbent assay (ELISA) measurements allows usage of linear models for data analysis

**DOI:** 10.1186/s12985-022-01804-3

**Published:** 2022-05-18

**Authors:** Thomas M. Lange, Maria Rotärmel, Dominik Müller, Gregory S. Mahone, Friedrich Kopisch-Obuch, Harald Keunecke, Armin O. Schmitt

**Affiliations:** 1 Breeding Informatics Group, University of Göttingen, Margarethe von Wrangell-Weg 7, 37075 Göttingen, Germany; 2 KWS SAAT SE & Co. KGaA, 37574 Einbeck, Germany; 3 Center of Integrated Breeding Research (CiBreed), University of Göttingen, Carl Sprengel-Weg 1, 37075 Göttingen, Germany

**Keywords:** Data analysis, Virus concentration, Serial dilution, Logistic regression, Generalised least squares model, Beet necrotic yellow vein virus, BNYVV

## Abstract

**Background:**

In research questions such as in resistance breeding against the *Beet necrotic yellow vein virus* it is of interest to compare the virus concentrations of samples from different groups. The enzyme-linked immunosorbent assay (ELISA) counts as the standard tool to measure virus concentrations. Simple methods for data analysis such as analysis of variance (ANOVA), however, are impaired due to non-normality of the resulting optical density (OD) values as well as unequal variances in different groups.

**Methods:**

To understand the relationship between the OD values from an ELISA test and the virus concentration per sample, we used a large serial dilution and modelled its non-linear form using a five parameter logistic regression model. Furthermore, we examined if the quality of the model can be increased if one or several of the model parameters are defined beforehand. Subsequently, we used the inverse of the best model to estimate the virus concentration for every measured OD value.

**Results:**

We show that the transformed data are essentially normally distributed but provide unequal variances per group. Thus, we propose a generalised least squares model which allows for unequal variances of the groups to analyse the transformed data.

**Conclusions:**

ANOVA requires normally distributed data as well as equal variances. Both requirements are not met with raw OD values from an ELISA test. A transformation with an inverse logistic function, however, gives the possibility to use linear models for data analysis of virus concentrations. We conclude that this method can be applied in every trial where virus concentrations of samples from different groups are to be compared via OD values from an ELISA test. To encourage researchers to use this method in their studies, we provide an R script for data transformation as well as the data from our trial.

**Supplementary Information:**

The online version contains supplementary material available at 10.1186/s12985-022-01804-3.

## Introduction

Since its invention in 1972, the enzyme-linked immunosorbent assay (ELISA) [1] has been used until today as a reliable tool to detect and quantify virus concentrations in humans [2–8], animals [9–11], and plants [12–18]. To quantify virus concentrations in a sample, double antibody sandwich ELISA (DAS-ELISA) is a common tool where an enzyme-linked antibody binds specifically to the coat protein of the virus, which again is bound to the surface of a microtiter plate by another specific antibody. Subsequently, a colourless substrate is given to each sample which is decomposed over time, leading to a lower transmittance of light. This reaction is catalysed by the enzyme [13, 19]. To measure the transmittance level of the sample at a certain time point, light with a certain wavelength can be send through the sample. It can then be measured via a sensor how much of the light was absorbed by the sample which results in an optical density (OD) value for each sample [20]. Due to the measurement of transmittance levels, the resulting ODs can only take on values within a certain interval. In case of the ELISA machine in this trial, this interval was from zero to four.

The numeric form of ODs makes it possible to compare the ODs of different groups of samples to find significant differences in protein concentrations of these groups. Such studies have been performed in the field of medicine [21–26], veterinary medicine [27], neuroscience [28, 29], pharmacology [30], and agriculture [31–34]. To find significant differences of ODs of different groups, analysis of variance (ANOVA) is the established standard. ANOVA can only be performed when normal distribution of response variables in each group can be assumed [35]. This requirement is rarely tested in data analysis of ODs, but if tested, it was rejected for at least one group [27, 29, 34].

Here, we present data from a trial where doubled haploid lines of sugar beet (*Beta vulgaris* L.) were grown in soil infested with *Beet necrotic yellow vein virus* (BNYVV) in different environments and harvested at different time points. Due to relatively large sample sizes per group and usage of genetically identical individuals, we show that the data of ODs per group are not normally distributed. This coincides with the observations from a similar trial [37] and explains the result of distribution tests in [27, 29, 34].

While performing the ELISA test, we were using a serial dilution of a sample with a high virus concentration on each of the ELISA plates. We visualise the relationship between virus concentration and OD and test different statistical models for mathematical description of the relationship. We show that a non-linear regression model with three parameters (inflection point, slope at inflection point, and asymmetry) as well as predefined values for bottom and top asymptotes could model the relationship best.

Moreover, we used the inverse of this model for data transformation of all measured OD values of the corresponding plate. We show that the transformation not only transferred the measurements on a linear scale but also led to a normal distribution of the transformed values per group. The transformed data, however, also show unequal variances of the groups. Due to these results, we discourage researchers to analyse data from an ELISA test using ANOVA. However, we show that the transformed data can be analysed using a generalised least squares (gls) model which allows for different variances of the groups.

Since the serial dilutions in the presented study were developed without knowing the absolute protein concentration of the parent solution, we conclude that this method can be applied in any experiment where protein concentrations of samples from different groups are to be compared via OD values from an ELISA test. Moreover, we provide an R script as well as the data from this trial as supplementary material to give researchers the possibility to transform raw data from an ELISA test according to our findings.

## Materials and methods

### Design of experiment

Seven doubled haploid lines of sugar beet were used in this trial, five of which were resistant towards rhizomania (KWS A, KWS B, KWS C, KWS D, KWS E) and two susceptible (KWS F, KWS G) according to [36]. One week after seeds were germinated in sterile soil, seedlings were transplanted into a soil sand mixture (40:60) with soil from Pithivier (France) to ensure the presence of BNYVV pathotype P. Plants were grown in two environments: Environment 1 contained 560 plants which were grown in a greenhouse at around 25$^{\circ }$ at day and 16$^{\circ }$ at night. Environment 2 contained 560 plants which were grown in a climate chamber at around 25$^{\circ }$ at day and 12$^{\circ }$ at night with a period of 18 hours of light per day.

One half of the plants from each environment were harvested after seven weeks (harvest time point 1) and the other half after ten weeks (harvest time point 2), respectively. During harvesting, lateral roots were separated from the root body and plant sap was extracted from the plants by pressing the harvested lateral roots. 50 μg of plant sap was given into 500 μg of buffer solution containing ${1.59} {\rm{g}}$ $\text {Na}_2\text {CO}_3$, ${2.93}{\rm{g}}$ $\text {NaHCO}_3$, and ${0.2}{\rm{g}}$ $\text {NaN}_3$ solved in ${1}{\rm{l}}$ distilled water. For every combination of genotype, harvest time point and environment, 40 plants were grown, thus, in total, 1120 plants were used in this trial.

The plants were grown in boxes, each containing 35 plants. In each box, 7 genotypes were planted with 5 repetitions each. The plants of one genotype were planted in rows and the rows were randomised. Each box was assigned to a harvest time point in a way that boxes were harvested in an alternating order. The samples were put onto the ELISA plates in the same order as they were standing in the greenhouse or climate chamber, thus, on each ELISA plate were samples of each harvest time point, but plants grown in the greenhouse and in the climate chamber were analysed on different ELISA plates. The determination of OD values was performed using the protocol in [37]. This protocol stipulates 90 min between adding the substrate to the samples and measuring the OD values using the Infinite F50® (Tecan Group AG, Männedorf, Switzerland) at a wavelength of 405 nm. This machine produces OD values between zero (theoretically minimal absorbance) and four (maximum absorbance). Moreover, this protocol includes four buffer controls on each 96 well plate to estimate the background noise of the machine.

### The serial dilution

To perform DAS-ELISA with all samples collected in the trial, eleven 96 well plates were used. On each 96 well plate, a serial dilution of twelve samples was analysed. To produce the serial dilution, a sample with a high virus concentration was produced (the “parent solution”) and its virus concentration was halved to produce the next sample of the serial dilution which has then been halved in its virus concentration to produce the next sample of the serial dilution, and so on. Though the virus concentration of the parent solution ($C_0$) is not known, the virus concentration of each sample *i* of the serial dilution can be put into a relationship with the virus concentration of the parent solution:

1 $$\begin{aligned} C_{i} = \frac{C_{0}}{2^{i}} \end{aligned}$$

where $C_i$ is the virus concentration of the *i*-th sample in the serial dilution.

### Statistical modelling of the serial dilution

The ODs of the samples in the serial dilution were compared to their relative virus concentration where the virus concentration of the last sample of the serial dilution was arbitrarily defined as $C_{11} = 1$. Thus, $C_{10}=2$, $C_{9}=4$, and so on until $C_0 = 2048$. If instead the dual logarithm of the relative virus concentrations is used, the difference between two successive samples in the serial dilution is 1. Thus, the ODs of each sample in the serial dilution were compared to the dual logarithm of the relative virus concentration ($\text {ld}(C_i)$).

To describe the relationship between ODs and the logarithmised relative virus concentration, we have used a five parameter logistic regression (5PL) model. The general structure of the 5PL model is given in Commo and Bot [38]. Commo and Bot [38], however, used the decadic logarithm in their calculations. Here, we use the dual logarithm since each sample in the serial dilution was created by halving the virus concentration of the previous sample in the serial dilution. Thus, the 5PL model in [38] was adapted as follows:

2 $$\begin{aligned} {\widehat{OD}}_{i} = B + \frac{T - B}{[1 + 2^{S \cdot (\text {ld}(I)-\text {ld}(C_{i}))}]^{A}} \end{aligned}$$

where ${\widehat{OD}}_i$ is the estimated OD value for the relative virus concentration $C_i$. This model is based on five parameters $\Theta = \{B, T, I, S, A\}$ where *B* is the background noise of the test or the OD that we would expect for a sample with a virus concentration of zero. *T* is the maximum OD that can be measured. *A* describes the asymmetry of the curve. *I* is the relative virus concentration ($C_i$) at the inflection point if $A = 1$. *S* is the slope at the inflection point.

Afterwards, we were interested if the complexity of the model can be optimised. Therefore, we have created one model where the asymmetry parameter was set to be one ($A=1$), one model where the slope was set to be one ($S=1$), and one model where the bottom asymptote was set to be zero ($B=0$). Furthermore, we have created one model where the top asymptote was set to be the technical limit of the machine ($T=tl$) and one model where the bottom asymptote of the machine was set to be the median of buffer controls for each 96 well plate ($B={\tilde{bc}}$).

Moreover, we were interested if multiple predefined parameters can further increase the quality of the model. Therefore, we have created a model where the top asymptote was set to be the technical limit of the machine and the bottom asymptote was set to be the median of the buffer controls for each 96 well plate ($B={\tilde{bc}}$ and $T=tl$). This will change Eq. 2 to

3 $$\begin{aligned} {\widehat{OD}}_{i} = {\tilde{bc}} + \frac{tl - {\tilde{bc}}}{[1 + 2^{S \cdot (\text {ld}(I)-\text {ld}(C_{i}))}]^{A}}. \end{aligned}$$

Equation 3 can be inverted such that the dual logarithm of the relative virus concentration ($\text {ld}(\widehat{C_i})$) can be calculated from the *i*-th OD value ($OD_i$). We present the equation of the inverse logistic regression in its logarithmised form for the sake of clarity.

4 $$\begin{aligned} \begin{aligned} \text {ld}(\widehat{C_{i}})&= \text {ld}(I) - \frac{\text {ld}((\frac{tl-{\tilde{bc}}}{OD_i-{\tilde{bc}}})^\frac{1}{A}-1)}{S} \\&= \text {ld} \left( \frac{{I}}{[(\frac{tl-{\tilde{bc}}}{OD_{i}-{\tilde{bc}}})^{\frac{1}{{A}}}-1]^{\frac{1}{{S}}}} \right) \end{aligned} \end{aligned}$$

*Parameter optimisation* For each plate each model was fitted to the data points of the serial dilution by optimising the parameters in the model such that the sum of squared errors (SSE) is minimised [39]:

5 $$\begin{aligned} SSE = \sum _{i=1}^n w_i (OD_i - \widehat{OD_i})^2 \end{aligned}$$

with $w_i$ being a general weights parameter defined as

6 $$\begin{aligned} w_i = \frac{1}{\text {ld}(C_i)} \end{aligned}$$

to ensure that the curve is not over fitting data points near the upper asymptote while poorly fitting data points near the bottom asymptote [38].

The models were fitted for each 96 well plate separately using the R function nlsLM from the minpack.lm package [40, 41]. The Levenberg-Marquardt algorithm was used to to minimise SSE [42, 43]. We have used nlsLM instead of nls or nls2 [44] which are based on the Gauss-Newton algorithm because of the robustness of the Levenberg-Marquardt algorithm and the function nlsLM even for poorly chosen start values [45]. Moreover, the function nlsLM also led to lower SSE values for the models than nls or nls2 (data not shown). Since only a positive correlation between virus concentration and OD is assumed, the following assumptions can be made about the model parameters and can be used as bounds of the parameters:

7 $$\begin{aligned} \begin{aligned} 0 \le B&< T \le tl\\ 0&< S\\ 0&< A \end{aligned} \end{aligned}$$

*Quality evaluation* For every serial dilution, twelve data points were supposed to be modelled with three to five parameters. Thus, there are a relatively large number of parameters in the models compared to the number of data points per serial dilution. Subsequently, the corrected Akaike information criterion (AICc) was used to evaluate the quality of the models since it is assumed to perform better than other quality parameters such as the Akaike Information Criterion in small samples where the quotient of observations (*n*) and parameters in the model ($m_j$) is smaller than 40 [46, 47]. The AICc of model *j* is defined as [48]

8 $$\begin{aligned} AICc_j = AIC_j + \frac{2(m_j+1)(m_j+2)}{n-m_j-2}. \end{aligned}$$

$AIC_j$ is defined as $AIC_j = -2\text {log}(L_j) + 2V_j$ where $L_j$ is the maximum likelihood for the candidate model *j* and $V_j$ is the number of parameters in model *j* [49, 50]. The calculation of AICc has been done with the R package MuMIn [47].

*Model selection and data transformation* The OD values were transformed using Eq. 4 for each 96 well plate. When transforming data using non-linear regression with top and bottom asymptote, only data in the interval of monotonic growth of the regression model can be transformed. If a sample did not contain any virus, its OD should result from the background noise of the machine. In this way, it could be lower or equal to the bottom asymptote set in the model. Due to this, no distinct virus concentration can be estimated for an OD value that is equal or smaller than the bottom asymptote. The same holds for ODs that equal the upper asymptote of the model. Thus, only ODs between the bottom and upper asymptote of model *j* could be transformed, other data had to be removed.

### Analysing data distributions

For each combination of genotype (7 levels), environment (2 levels), and harvest time point (2 levels), the data distribution was analysed by testing for skewness using the D’Agostino $K^2$ test [51, 52] and testing for non-normality using Shapiro-Wilk test [53]. We performed the D’Agostino $K^2$ test using the R function agostino.test from the R package moments with two-sided alternative hypothesis that the data are skewed [54]. If the *p* value from the D’Agostino test was smaller than 0.05, it was assumed that the data distribution was skewed. We performed the Shapiro-Wilk test with the base R function shapiro.test with default settings. We assumed non-normality of the data if $p < 0.05$ [55]. Moreover, we tested if the variances of the 28 groups differed significantly. Therefore, we used Levene’s test of equality of variances [56]. To perform Levene’s test, the R function levene.test from the package lawstat was used [57]. With default settings, this function uses the modified Brown-Forsythe Levene-type procedure [58]. We assumed unequal variances if $p < 0.05$.

### Data analysis

To estimate the effect of the environment and simultaneously the effect of the harvest time point on ODs and transformed data, a gls model was used that allowed for different variances per group, following suggestions in [59, 60]. Therefore, a gls model was created using the R function gls where the variance structure was integrated using the R function varIdent. Both functions are from the package nlme [61]. Afterwards, differences between the models were examined using the base R function anova with the gls model. The gls models included all possible combinations of interaction terms of the three variables. The model formulation was set to be

9 $$\begin{aligned} Y_{ijkl} = \mu + \alpha _{i} + \beta _{j} + \gamma _{k} + (\alpha \beta )_{ij} + (\alpha \gamma )_{ik} + (\beta \gamma )_{jk} + (\alpha \beta \gamma )_{ijk} + \varepsilon _{ijkl} \end{aligned}$$

where $\mu$ is the overall mean response, $\alpha _{i}$ is the effect due to the *i*-th level of the genotype, $\beta _{j}$ is the effect due to the *j*-th level of the environment, and $\gamma _{k}$ is the effect due to the *k*-th level of the harvest time point. Moreover, $(\alpha \beta )_{ij}$ is the effect due to any interaction between the *i*-th level of the genotype and the *j*-th level of the environment, $(\alpha \gamma )_{ik}$ is the effect due to any interaction between the *i*-th level of genotype and the *k*-th level of the harvest time point, $(\beta \gamma )_{jk}$ is the effect due to any interaction between the *j*-th level of the environment and the *k*-th level of the harvest time point, and $(\alpha \beta \gamma )_{ijk}$ is the effect due to any interaction between the *i*-th level of genotype, the *j*-th level of environment, and the *k*-th level of the harvest time point. The response variable ($Y_{ijkl}$) represents either the OD value or the transformed data. $\varepsilon _{ijkl}$ is an error term for the *l*-th subject with genotype *i*, environment *j*, and harvest time point *k* and was assumed to be

10 $$\begin{aligned} \varepsilon _{ijkl} \sim N(0, \sigma ^2_{ijkl}). \end{aligned}$$

### Robustness of the method

We were moreover interested in the robustness of this method. Therefore, we have used a cross validation where we have estimated the parameters of the model in ten of the eleven plates. Subsequently, we have calculated the median of the ten estimations for the parameters and have transformed the data on the eleventh plate using the median from the parameter estimations from the other ten plates. Afterwards, the results from both transformations were compared regarding the normal distribution of the resulting transformed data and regarding the number of data points that were outside the monotonic growth region of the model and could, thus, not be transformed.

## Results

### Statistical modelling and model selection

**Fig. 1:**
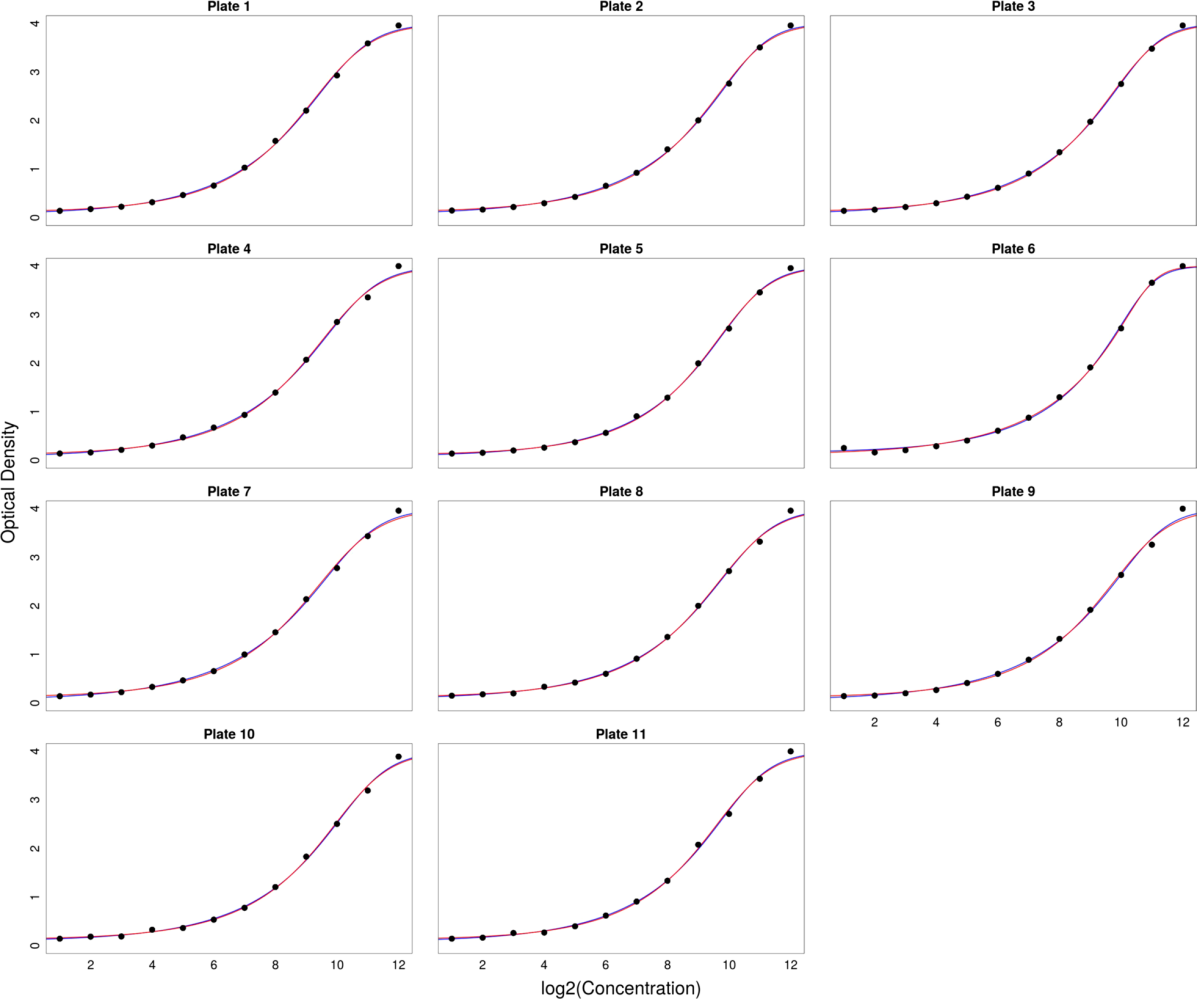
The eleven serial dilutions used in this trial and the fitted curves. The dual logarithm of the relative concentration is displayed on the X axis, the corresponding OD value is displayed on the Y axis. Blue line: 5PL model, red line: 5PL model with predefined values for top and bottom asymptote, black dots: observed values

As described above, a serial dilution with 12 samples was used on each of the eleven 96 well plates. Moreover, four buffer controls were used on every 96 well plate. Each serial dilution was modelled using the logistic regression models as described above. The serial dilutions as well as the corresponding regression models can be seen in Fig. 1.

One can see in Fig. 1 that the relationship between OD and $\text {ld}(C)$ is non-linear. Moreover, one can see in Fig. 1 that the 5PL model as well as the 5PL model with predefined values for bottom and top asymptote were able to model the data points of the serial dilution very well. Both lines in Fig. 1 are displaying a non-linear relationship. Based purely on visual examination, it is difficult to see a difference between the two models in Fig. 1. It is reasonable to assume that if the models are very similar, the lower complexity of the model with predefined values will lead to a smaller AICc.

This assumption is confirmed by the AICc values for each combination of serial dilution and regression model, shown in Table 1. The mean of the AICc values from the 5PL model where no parameters have been predefined is $-16.4$. One can see that the AICc increased dramatically when the parameters for asymmetry or slope have been predefined as 1. This shows that these parameters are increasing the quality of the model if they can be optimised freely. This coincides with results from [39].

If the upper or bottom asymptote have been predefined on the other hand, the mean AICc was decreased. One can see that in many cases, the AICc was decreased if the bottom asymptote has been predefined as zero or as the median of the buffer controls. The same holds if the upper asymptote has been predefined as the technical limit of the machine. Basing on these results, we have created one more model in which the upper and bottom asymptote have been predefined simultaneously, the upper asymptote as the technical limit and the bottom asymptote as the median of buffer controls.

**Table 1:** AICc of the 5PL model with different predefined values for the eleven serial dilutions as well as the mean AICc for all eleven serial dilutions

	Predefined parameters						
	none	$A=1$	$S=1$	$B=0$	$B={\tilde{bc}}$	$T=tl$	$B={\tilde{bc}}$
							$$T=tl$$
**Plate**	**AICc of the model**						
1	-22.714	-8.724	-6.421	-24.688	-26.267	-31.514	-32.552
2	-25.508	-4.557	-2.636	-26.232	-26.789	-34.308	-33.074
3	-30.735	-4.473	-2.052	-26.85	-29.595	-39.535	-35.881
4	-11.73	-4.226	-3.198	-17.608	-17.548	-20.53	-23.833
5	-19.368	-6.963	-3.423	-18.951	-25.138	-28.168	-31.423
6	-5.105	0.005	4.449	-1.516	-12.002	-13.905	-18.288
7	-17.987	-6.82	-6.319	-23.749	-20.692	-26.787	-26.978
8	-13.647	-6.684	-5.212	-16.764	-20.742	-22.447	-27.028
9	-9.264	-3.486	-2.567	-16.195	-14.567	-18.064	-20.852
10	-12.951	-6.583	-4.548	-14.438	-19.944	-21.751	-26.23
11	-10.923	-4.824	-2.335	-15.069	-17.42	-19.723	-23.705
mean	-16.4	-5.2	-3.1	-18.4	-21	-25.2	-27.3

Based on the results shown in Table 1, we have selected the 5PL model with predefined values for the upper and bottom asymptote as the best statistical model since it has shown the smallest AICc for nine of the eleven serial dilutions and provided the smallest AICc on average. Thus, data were transformed using Eq. 4. Parameters $\Theta = \{I, S, A\}$ were optimised freely to minimise the SSE (Eq. 5) for the serial dilution on each plate.

From the 1120 plants grown in this trial, 816 ODs could be measured in the ELISA test. From these ODs, four were at the technical limit of the machine ($OD=4$) and five were smaller than the median of the buffer controls of the plate. Thus, these 9 ODs could not be transformed and were removed from the analysis. Subsequently, 807 measurements could be transformed using the inverted logistic regression model and analysed further. Afterwards, sample sizes of the 28 groups reached from $n=3$ to $n=40$. Since we considered a sample size of $n=3$ as too small, this group (KWS D, environment 2, harvest time point 2) had to be removed from the data as well. The other groups showed a sample size of $n\ge 9$.

### Analysing data distributions

Each combination of genotype, environment and harvest time point was considered a distinct group and data distribution of OD values and transformed data for each group were analysed using the D’Agostino $K^2$ test [51, 52] and the Shapiro-Wilk test [53]. The resulting *p* values are given as an Additional file 3. The null hypothesis of symmetrical data distribution was rejected if the *p* value from the D’Agostino $K^2$ test was smaller than 0.05 and the null hypothesis of normally distributed data was rejected if the *p* value from the Shapiro-Wilk test was smaller than 0.05, respectively. Moreover, Levene’s test was used to check for equal variances. If the *p* value from Levene’s test was smaller than 0.05, unequal variances were assumed [56].

The D’Agostino test with the OD values as response variable led to *p* values smaller than 0.05 for 21 of the 27 groups under investigation. Four of the six groups that showed a *p* value greater than 0.05 belonged to the susceptible genotype KWS G. The Shapiro-Wilk test with the OD values as response variable led to *p* values smaller than 0.05 for 24 of the 27 groups under investigation. From the six groups that showed a *p* value greater than 0.05 with the D’Agostino test, only two groups also showed a *p* value greater than 0.05 with the Shapiro-Wilk test and only one of them belongs to to the genotype KWS G. It can be assumed that the data distribution of OD values is symmetrical for susceptible genotypes, thus, for samples with high virus concentrations but nevertheless being non-normally distributed.

For the *p* values from the D’Agostino test with the transformed data as response variable, only four showed a *p* value smaller than 0.05. Similar results can be noticed for the *p* values from the Shapiro-Wilk test with the transformed data as response variable. In this case, only two groups resulted in a *p* value smaller than 0.05. The transformation has reduced the number of significant results for distribution tests indicating non-normal data distribution from 21 to four groups regarding the D’Agostino $K^2$ test and from 24 to two groups, regarding the Shapiro-Wilk test.

Levene’s test led to a *p* value of $4 \cdot 10^{-31}$ with the OD values as response variable and to a *p* value of $3 \cdot 10^{-13}$ with the transformed values as response variable. One can see that the *p* value was increased due to the transformation but with a significance threshold of $\alpha =0.05$, the assumption of equal variances must be rejected for both response variables.

### Data analysis

Regarding the results from the distribution tests with the transformed data, it can be assumed that the transformed data are normally distributed but show unequal variances. Thus, a gls model was used which incorporated unequal variances. Afterwards, an ANOVA output can be produced using the base R function anova. The results from analysis suggest that the genotype had a significant effect on the result as well as the environment (Table 2). The harvest time point had no significant effect. Nevertheless, the interaction between genotype and harvest time point was significant as well as the interaction between genotype and environment. The interaction between harvest time point and environment as well as the interaction between all three variables were not significant.

**Table 2:** Results from the gls model with transformed data as response variable

	numDF	F-value	p-value
(Intercept)	1	9481.088	<.0001
Genotype	6	255.132	<.0001
HarvestTimePoint	1	0.011	0.9163
Environment	1	4.181	0.0412
Genotype:HarvestTimePoint	6	4.195	0.0004
Genotype:Environment	6	5.714	<.0001
HarvestTimePoint:Environment	1	0.022	0.8824
Genotype:HarvestTimePoint:Environment	6	0.885	0.5051

### Robustness of the method

Moreover, a cross validation was used to analyse the robustness of the method. Therefore, the transformation was done with ten of the eleven serial dilutions and the median of each parameter was calculated. Subsequently, the data on the eleventh plate were transformed with these medians and with the serial dilution on the eleventh plate itself. Afterwards, the results were compared regarding applicability and data distribution per group.

As described above, the data transformation with the 5PL model and predefined values for the upper and bottom asymptote resulted in four data points that were at the technical limit of the machine and could, thus, not be transformed as well as five data points that were smaller than the median of the buffer controls and could not be transformed for that reason. The transformation resulted in data that were mostly normally distributed with five groups where the D’Agostino $K^2$ test resulted in a *p* value smaller than 0.05 and with two groups where the Shapiro-Wilk test resulted in a *p* value smaller than 0.05. The transformation via this cross-validation led to similar results. The D’Agostino $K^2$ test resulted again five times in a *p* value smaller than 0.05 and the Shapiro-Wilk test resulted two times in a *p* value smaller than 0.05. Nevertheless, the cross-validation resulted in 28 data points that were smaller than the median of the buffer controls and could, thus, not be transformed.

## Discussion

In research questions where the effect of multiple variables on the response variable is of interest, ANOVA is the established standard for data analysis even though it requires assumptions to be made about the distribution of the response variable. We show that in the present study, these requirements were not met for the ODs measured in an ELISA test. Similar findings were made in experiments where protein concentrations were measured with ELISA and the normality assumption was tested. None of these tests suggested a normal distribution [27, 29, 34].

Furthermore, we show that the relationship between ODs and virus concentration in this trial was non-linear. Assuming that the relationship between protein concentration and OD always follows a non-linear relationship, it could be concluded that although the virus concentration might follow a normal distribution, the data distribution of ODs might not. If normal distribution of the response variable is not given, simple methods for data analysis such as ANOVA cannot be used to reliably analyse the data [62]. Thus, ANOVA should not be used to analyse the raw ODs from an ELISA test. Due to our experience, we presume that a simple transformation of the data (i.e. decadic logarithm of the OD values) is not sufficient to transform the data into normally distributed data. Following this assumption, some researchers use non-parametric hypothesis tests (such as *U* test or Kruskall-Wallis test) to analyse ODs from an ELISA test [22, 27, 29, 63]. Nevertheless, non-parametric hypothesis tests have less test power than their parametric equivalents which might explain why the majority of researchers use parametric hypothesis tests such as ANOVA or *t* test to analyse ODs from an ELISA test [24, 26, 28, 34, 64–66].

Here, we present an alternative to these methods by transforming the ODs with an inverse logistic function where parameters were fitted based upon serial dilutions. The OD values were on a non-linear scale, i.e. an OD value of four cannot be assumed to be twice an OD value of two. The transformed data, however, are on a linear scale, thus, a transformed value of four can be assumed to be twice a value of two. We show that the transformed data are normally distributed per group but still do not provide equal variances. This means that linear models can be used if an appropriate variance structure is integrated into the model. Thus, if only one variable is analysed, Welch’s ANOVA can be applied, and if multiple variables are analysed, a gls model can be used which incorporates a variance structure with a different variance per group. In this way, linear models can be used to reliably analyse data from an ELISA test.

We assume that the presented transformation can be applied in any experiment where ELISA is used to differentiate between protein concentrations since we have performed the data transformation with a serial dilution for which we have not known the distinct virus concentrations in the sample but only referred each sample’s virus concentration to the parent solution ($C_0$) in the serial dilution. Thus, this method can be applied in any ELISA trial even if a serial dilution with known protein concentrations cannot be produced.

Moreover, we analysed if the complexity of the 5PL model can be optimised by predefining some of the parameters. Since the number of samples that can be analysed on an ELISA plate is limited, the number of samples in a serial dilution is limited, too. Thus, one has to assume that relatively few data points can only be available for modelling the serial dilution and, thus, a reduction of the number of parameters in the logistic regression model will be beneficial. We found that predefining the asymmetry parameter or the slope parameter decreased the quality of the model dramatically. Similar findings regarding the asymmetry parameter were published in [39]. We found that the upper and bottom asymptote of the model can be predefined as the median of the buffer controls for the bottom asymptote and as the technical limit of the machine for the upper asymptote. Nevertheless, it should be pointed out that this method can only be applied if the protocol of the ELISA test indicates that the upper asymptote of the serial dilution can approximate the technical limit of the machine. If there is too little time between adding the substrate and measuring the ODs, the upper asymptote will form its plateau below the technical limit of the machine. In this case, the technical limit of the machine should not be set as upper asymptote of the logistic model.

Finally, we have used a cross-validation to analyse the robustness of this method. We found that data that were transformed in this way can also be assumed to be normally distributed with five of the 27 groups under investigation leading to a *p* value smaller than 0.05 with the D’Agostino $K^2$ test and four of the 27 groups leading to a *p* value smaller than 0.05 with the Shapiro-Wilk test. Nevertheless, 28 data points could not be transformed in the cross validation, showing the necessity of measuring the background noise on each ELISA plate. In this case it can be argued that once a model for data transformation is derived, it can be used for transformation of data from an ELISA test without running a serial dilution on each ELISA plate. In this way, resources could be saved. Nevertheless, it must be kept in mind that this assumption only holds if serial dilutions are very similar to each other as it was the case for the eleven serial dilutions in this trial. Therefore, high amount of precision is necessary which has to be kept over the course of multiple trials. It can be argued that such a precision might be reached through automatisation of work processes in the performance of the ELISA test but further research would be necessary to answer this question.

## Conclusion

In research questions where the effect of multiple variables on the response variable is of interest, ANOVA is the established standard for data analysis. One requirement of ANOVA is the normal distribution of the response variable per group. This makes non normality a common problem in many situations. We show that in this trial, the ODs from the ELISA test were not normally distributed which might be due to the non-linear relationship between virus concentration and OD. To model a non-linear relationship with two plateaus, logistic regression models are an appropriate choice.

We show that a logistic regression model with predefined values for the bottom and top asymptote as well as three free parameters which can be estimated using a serial dilution can model the relationship between virus concentration and OD values accurately. Using the inverse of the logistic regression model, data can be transformed to estimate the virus concentration for every OD value that has been measured in the ELISA test. Furthermore, we show that though the measured OD values are not normally distributed, the estimated virus concentrations are normally distributed and can be analysed using linear models.

Since the serial dilutions were prepared without knowing the absolute protein concentrations in these samples, we anticipate that this method can be applied in every experiment where protein concentrations of samples from different groups are to be compared via ODs from an ELISA test.

## Supplementary Information

**Additional file 1**. R script for transformation of optical density values from an ELISA test. The script expects a column sample that contains the string bc for each buffer control, the string sample for each sample in the trial and the string serialdilution_ and the corresponding number of the sample of the serial dilution for each sample of the serial dilution. The R script is also available at https://github.com/tmlange/TransformELISA.

**Additional file 2**. Data from the presented trial with six columns. One column describes the sample, one column describes the ELISA plate, and one column contains the corresponding optical density value from the ELISA test. Three more columns describe the factors to be analysed in the trial: The genotype, the environment, and the harvest time point.

**Additional file 3**. Description of the 28 groups via sample size (*n*), *p* value from the D’Agostino *K*^2^ test (*pA*) for each response variable, and p value from the Shapiro-Wilk test (*pS*) for each response variable. For all analyses, OD values (*OD*) and transformed data (*C*) were used as response variables. For the group with genotype KWS D grown in environment 2 and harvested at time point 2, only 3 data points were available. Thus, for this group neither Shapiro-Wilk test nor D’Agostino *K*^2^ test were performed.

## Data Availability

The data set supporting the conclusions of this article is included within the article (and its Additional file 1, 2, 3).

## Abbreviations

5PL: Five parameter logistic regression
AICc: Corrected Akaike information criterion
ANOVA: Analysis of variance
BNYVV: *Beet necrotic yellow vein virus*
ELISA: Enzyme-linked immunosorbent assay
gls: Generalised least squares
OD: Optical density
SSE: Sum of squared errors
